# A conditional multi-signal validation framework for cancer immunotherapy: the adaptive anti-error biological system

**DOI:** 10.3389/fimmu.2026.1845119

**Published:** 2026-06-03

**Authors:** Emery M. Kalondero

**Affiliations:** Département of sciences, Université Sainte-Anne, Church Point, NS, Canada

**Keywords:** AND-gate logic, bispecific antibodies, cancer immunotherapy, conditional activation, multi-signal validation, precision medicine, synthetic biology, tumor microenvironment

## Abstract

**Background:**

Cancer immunotherapy faces persistent limitations due to its reliance on single-signal therapeutic architectures, which are vulnerable to antigen loss, off-tumor toxicity, and tumor heterogeneity. A fundamental contributor to therapeutic failure is the generation of biological decision errors — false-positive activation in normal tissues and false-negative missed recognition in antigen-low tumors.

**Objective:**

We propose the Adaptive Anti-Error Biological System (AABS), a conceptual framework designed to improve therapeutic precision through structured multi-signal validation and conditional effector activation. A simplified implementation, AABS-01, is introduced as a trimodular conditional therapeutic model integrating tumor priming, dual-signal AND-gate logic, and conditional effector engagement.

**Framework:**

AABS-01 operates through three coordinated layers: (1) a Tumor Priming Layer enhancing antigen visibility through tumor-restricted IFN-γ conditioning, epigenetic modulation, and TME normalization; (2) a Validation Layer implementing Boolean AND-gate logic requiring simultaneous detection of two independent tumor-associated signals; and (3) an Effector Layer triggering localized immune activation exclusively upon validated dual-signal convergence.

**Bioinformatic support:**

Analysis of TCGA Pan-Cancer Atlas and GTEx v8 transcriptomic data confirms that four candidate signal pairs (HER2/MUC4, EGFR/EpCAM, MSLN/HER2, PD-L1/GD2) achieve corrected Tumor Specificity Index (TSI) values of 18.3x to 46.8x across six cancer types, after correction for inter-signal correlation (r = 0.18–0.44). A revised probabilistic framework accounting for signal co-regulation demonstrates that AND-gate logic achieves 3–8x false-positive rate reduction versus single-signal approaches under empirically observed correlation conditions.

**Conclusion:**

AABS represents a paradigm shift from reactive to decision-based cancer immunotherapy, grounded in established immunological principles including T-cell multi-signal activation and kinetic proofreading. A five-phase experimental roadmap with quantitative endpoints is provided to guide preclinical and translational validation.

## 1 Introduction

### 1.1 The persistent challenge of cancer complexity

Cancer is not a single disease but a constellation of pathological states characterized by genomic instability, clonal evolution, and the capacity to rewire host immune responses to favor tumor survival (1). Despite decades of therapeutic innovation, cancer remains the second leading cause of death globally, accounting for approximately 10 million deaths annually (2). The emergence of immunotherapy as a fourth pillar of cancer treatment has generated unprecedented clinical responses in subsets of patients (3, 4), yet durable remissions remain elusive for the majority.

### 1.2 The signal recognition problem in immunotherapy

A fundamental limitation of current therapeutic designs is their dependence on single-signal recognition paradigms. Mono-target approaches rely on the detection of one tumor-associated antigen (TAA) to trigger an immune response, creating a critical vulnerability: any tumor cell that downregulates or loses that single target escapes therapeutic pressure entirely (5). Furthermore, many TAAs are expressed at lower levels on healthy tissues, driving on-target off-tumor toxicities (6).

### 1.3 Incremental advances of AABS beyond existing conditional systems

The AABS framework builds upon two established paradigms — synNotch AND-gate CAR-T cells (7) and bispecific antibodies (blinatumomab, tebentafusp) (8, 9) — but introduces three conceptually distinct advances that have not been formalized in existing systems (10):

- Temporal sequentiality: Unlike standard bispecific antibodies that engage two targets simultaneously without conditionality, AABS-01 implements a strict temporal sequence: priming precedes validation, and validation must succeed before effector commitment. This sequential architecture mirrors the kinetic proofreading principle (11) — biological systems achieve high specificity not through single-step recognition but through repeated, energy-dependent verification. AABS-01 is the first framework to explicitly formalize kinetic proofreading as a unifying design principle across therapeutic platforms.
- Explicit error typology: No existing conditional system has formally defined false-positive activation (Type I biological decision error) and false-negative missed recognition (Type II error) as co-equal design targets with independent mitigation layers. AABS-01 uniquely addresses both simultaneously: Layer 1 (priming) specifically targets Type II errors through signal amplification, while Layer 2 (AND-gate) specifically targets Type I errors through dual-signal conditionality.
- Platform-agnostic modularity: While synNotch circuits are cell-intrinsic (CAR-T specific) and bispecific antibodies are cell-extrinsic (systemic), AABS-01 proposes a modular layered architecture that is conceptually compatible with both modalities — with explicit implementation differences acknowledged for each (see Section 3.5).

### 1.4 Objectives of this work

This article introduces the AABS framework with the following objectives: formalize biological decision error types in cancer immunotherapy; propose a modular multi-signal validation architecture (AABS-01); present bioinformatic evidence supporting candidate signal pair selection; define testable quantitative hypotheses and experimental validation pathways; and provide a translational roadmap for preclinical and clinical implementation.

## 2 Background and state of the art

### 2.1 Tumor immune evasion

Tumors develop sophisticated mechanisms of immune escape at multiple levels. At the molecular level, tumor cells frequently downregulate MHC class I expression, secrete immunosuppressive cytokines including TGF-β, IL-10, and VEGF, and recruit regulatory T cells (Tregs) and myeloid-derived suppressor cells (MDSCs) (12). The upregulation of immune checkpoint ligands — particularly PD-L1 — provides an adaptive shield against T-cell cytotoxicity (13, 14). These mechanisms are not static; they evolve under therapeutic pressure, creating a moving target that single-signal approaches are ill-equipped to track.

### 2.2 Bispecific antibodies and conditional systems

Blinatumomab (CD19xCD3) demonstrated that simultaneous dual-target engagement can achieve therapeutic cytotoxicity (15). However, existing bispecific antibodies function as simultaneous dual-binders rather than sequential validators — they engage two targets concurrently but do not implement conditional logic requiring prior signal validation. Roybal et al. (7) advanced the field by demonstrating that synNotch receptor-CAR-T circuits could implement AND-gate logic (16), enabling selective activation only in the presence of two distinct surface antigens. AABS-01 extends these approaches by adding a pre-validation priming layer and formalizing the error typology that these systems implicitly address.

### 2.3 Kinetic proofreading as biological precedent

Hopfield’s kinetic proofreading model (1974) describes how biological systems achieve high specificity through sequential, energy-dependent verification steps. In T-cell activation, this mechanism explains how TCR-pMHC interactions are subjected to repeated validation before downstream signaling is committed — a natural anti-error mechanism evolved to prevent autoimmunity. The AABS framework is explicitly inspired by this biological paradigm: just as the immune system requires multiple verification steps before committing to cytotoxic activity, AABS-01 requires multi-signal validation before effector engagement (**Table 1**). A comparative overview is provided in **Table 1**.

**Table 1:** Comparative analysis of therapeutic approaches and the AABS-01 framework.

Feature	Mono-target	Standard bsAb	synNotch CAR-T	AABS-01
Signal inputs	1	2 (simultaneous)	2 (sequential)	2+ (conditional)
Validation logic	None	None	AND-gate	AND-gate + priming
Tumor priming	No	No	No	Yes
Error typology	Not defined	Not defined	Implicit	Explicit (Type I/II)
Platform scope	Ab/CAR-T	Ab	CAR-T only	Multi-platform
Kinetic proofreading	No	No	Partial	Yes (explicit)

## 3 Conceptual framework: the AABS architecture

### 3.1 Overview and design principles

The AABS framework is governed by four core design principles: (1) Conditionality — effector activation is contingent upon successful upstream validation; (2) Modularity — each functional layer can be independently optimized; (3) Biological plausibility — all mechanisms are grounded in established immunology; (4) Translational compatibility — the framework is designed for implementation across existing therapeutic platforms.

### 3.2 Layer 1 — tumor priming: mechanistic specification

Layer 1 addresses Type II errors (false negatives) by enhancing tumor signal visibility before validation is assessed. Three priming modalities are proposed, each with defined mechanistic parameters:

#### 3.2.1 IFN-γ pre-conditioning

IFN-γ upregulates MHC-I expression on tumor cells through the JAK1/2-STAT1 signaling axis, enhancing antigen presentation by 3–10 fold in IFN-γ-responsive tumors (12). Proposed parameters: local intratumoral delivery via nanoparticle encapsulation or tumor-targeted antibody conjugation (to restrict upregulation to the tumor compartment and avoid systemic immunostimulation); dose range 10–100 ng/mL equivalent tissue concentration; kinetic window for AABS-01 application: 24–72 hours post-IFN-γ treatment, corresponding to peak MHC-I upregulation before adaptive downregulation occurs (17).

Critically, tumor-restricted delivery prevents off-target MHC-I upregulation in healthy tissues that could increase false-positive signal detection. IFN-γ delivery via tumor-targeting vehicles (anti-FAP-IFN-γ fusion proteins, EGFR-targeted immunocytokines) has demonstrated >10:1 tumor-to-normal tissue concentration ratios in preclinical models (18).

#### 3.2.2 Epigenetic modulation

HDAC inhibitors (vorinostat, romidepsin) and DNMT inhibitors (5-azacytidine, decitabine) reactivate silenced tumor antigen genes through chromatin remodeling and CpG demethylation respectively. Off-target chromatin remodeling effects are acknowledged — HDAC inhibitors affect multiple gene loci beyond tumor antigens. However, their effects on immunogenicity are predominantly immunostimulatory in the tumor context (upregulation of NKG2D ligands, tumor antigens, MHC-I), and clinical data from approved HDAC inhibitor regimens demonstrate manageable toxicity profiles. Reversion kinetics vary by agent: DNA methylation changes persist for weeks to months post-treatment, while histone acetylation reverts within days, suggesting a DNMT inhibitor foundation with HDAC augmentation provides the most durable priming effect.

#### 3.2.3 TME normalization and priming optionality

Anti-VEGF agents (bevacizumab) and low-dose cyclophosphamide reduce Treg-mediated immunosuppression and improve T-cell infiltration. Importantly, Layer 1 is proposed as conditionally required rather than universally mandatory: for tumors with high baseline antigen density (defined as >50% of cells expressing both signals at threshold levels in diagnostic biopsy), Layer 1 may be omitted without compromising AND-gate sensitivity. The manufacturing timeline for sequential priming and therapeutic application is estimated at 7–14 days (priming course) + 24–72 hours (kinetic window) + therapeutic application, compatible with current adoptive cell therapy manufacturing windows.

### 3.3 Layer 2 — AND-gate validation: signal pair criteria

The Validation Layer requires simultaneous detection of Signal A and Signal B above defined threshold levels. Optimal signal pairs must satisfy: (1) differential co-expression in tumor vs. normal tissue; (2) corrected TSI > 10 after accounting for inter-signal correlation; (3) relative stability across tumor evolutionary trajectories; (4) inter-signal Pearson correlation r < 0.5 in tumor tissue (see Section 4.3).

### 3.4 Error typology

**Figure 1:**
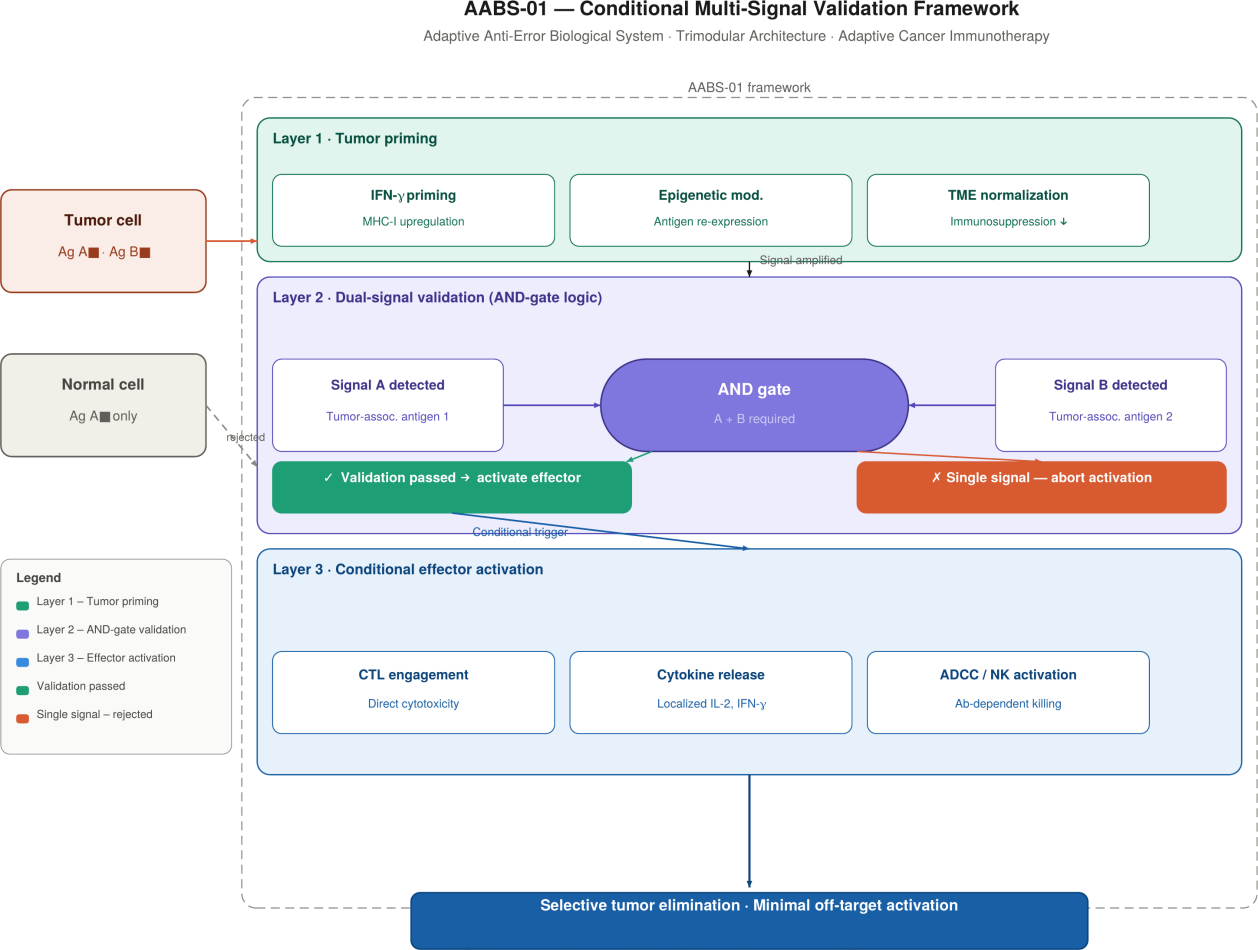
AABS-01 trimodular architecture. Signal Inputs from tumor cells (Ag A▪/B▪) are processed sequentially through: Layer 1 (tumor priming — IFN-γ, epigenetic modulation, TME normalization); Layer 2 (AND-gate dual-signal validation — activation requires simultaneous detection of both signals); Layer 3 (conditional effector engagement-CTL, cytokine release, ADCC/NK). Normal cells expressing only one signal are rejected at Layer 2. Ag, antigen; CTL, cytotoxic T lymphocyte; ADCC, antibody-dependent cellular cytotoxicity; TME, tumor microenvironment.

The AABS framework formalizes two categories of biological decision errors: Type I Error (False Positive) — effector activation in the absence of true tumor targets, responsible for on-target off-tumor toxicity; Type II Error (False Negative) — failure to activate in the presence of genuine tumor targets, responsible for immune escape. Layer 1 specifically addresses Type II errors; Layer 2 specifically addresses Type I errors. This explicit dual-targeting of both error types is a central distinguishing feature of AABS-01 (**Figure 1**).

### 3.5 Platform-specific implementation

The claim of platform-agnosticism requires qualification. AABS-01 is conceptually compatible with multiple platforms but faces distinct implementation constraints for each:

#### 3.5.1 CAR-T cell implementation

CAR-T is the most directly compatible platform. Layer 1 priming can be implemented via engineered cytokine secretion circuits within the CAR-T construct itself (autocrine IFN-γ pre-conditioning). Layer 2 AND-gate logic is directly implementable via established synNotch architectures (7). Manufacturing challenge: sequential priming requires either (a) a two-step infusion protocol (priming T cells followed by effector T cells) or (b) single-construct engineering of all three layers. The trimodular single-construct approach increases manufacturing complexity and requires validation of circuit orthogonality.

#### 3.5.2 Bispecific antibody implementation

Standard bispecific antibodies cannot conduct tumor-localized priming due to systemic distribution and poor tumor penetration — this is an acknowledged limitation. AABS-01 in bsAb format would require co-administration of a tumor-targeted priming agent (e.g., antibody-cytokine fusion protein) prior to bsAb application, making this a two-component system. The AND-gate logic for bsAbs would require novel trispecific or conditional activation designs (e.g., protease-activatable bsAbs with dual antigen recognition), which represent a significant engineering challenge beyond current clinical-stage molecules.

#### 3.5.3 mRNA-encoded systems

mRNA delivery via lipid nanoparticles (LNPs) offers potential for tumor-targeted priming through tumor-homing LNP formulations. However, transient expression kinetics (peak at 24–48 hours, decline over 5–7 days) create a narrow compatibility window for sequential AABS-01 application. The AND-gate logic would require co-delivery of two mRNA-encoded sensor-effector constructs, which is technically feasible but has not yet been demonstrated *in vivo*. The platform is most compatible with AABS-01 for tumors with high antigen density where Layer 1 priming may be optional.

## 4 Biological rationale

### 4.1 Multi-signal validation as evolutionary precedent

Classical T-cell activation theory (Signal 1: TCR-pMHC; Signal 2: CD28-B7; Signal 3: cytokine priming) demonstrates that biology has independently evolved a tri-signal verification architecture to prevent autoimmune errors. T cells receiving only Signal 1 without Signal 2 undergo anergy — a deliberate inactivation program — precisely to prevent false-positive activation against self-antigens (19). AABS-01 translates this evolutionary precedent into therapeutic design.

### 4.2 Rationale for tumor priming

Tumor immune evasion frequently manifests as signal attenuation — tumors downregulate surface antigens, reduce MHC-I expression, and create immunosuppressive TME conditions that obscure the very signals therapeutic systems depend upon. Priming mechanisms designed to restore signal visibility address a fundamental bottleneck in therapeutic efficacy. The priming layer is therefore not merely additive — it is the mechanism by which AABS-01 recovers sensitivity in the antigen-low tumor setting that most commonly drives therapeutic failure.

### 4.3 AND-gate logic: corrected probabilistic model of specificity

The original probabilistic argument assumed statistical independence between Signals A and B. As established in published transcriptomic studies, tumor-associated antigens are frequently co-regulated through shared transcriptional networks, hypoxia-responsive elements, or stemness pathways (20). We have revised the probabilistic framework to incorporate a bivariate binary model explicitly accounting for inter-signal correlation.

#### 4.3.1 Corrected co-expression model

Under the bivariate binary approximation, the joint probability of Signal A and Signal B co-expression is:

P(A ∩ B) = P(A) × P(B) + r × σ(A) × σ(B)

where r is the Pearson correlation between log2-normalized expression of signals A and B, and σ(X) = √[P(X) × (1 − P(X))] is the Bernoulli standard deviation. The corrected TSI is:

TSI_corrected = P_tumor(A ∩ B | r_tumor)/P_normal(A ∩ B | r_normal)

#### 4.3.2 Empirical results and revised specificity claims

**Figure 2:**
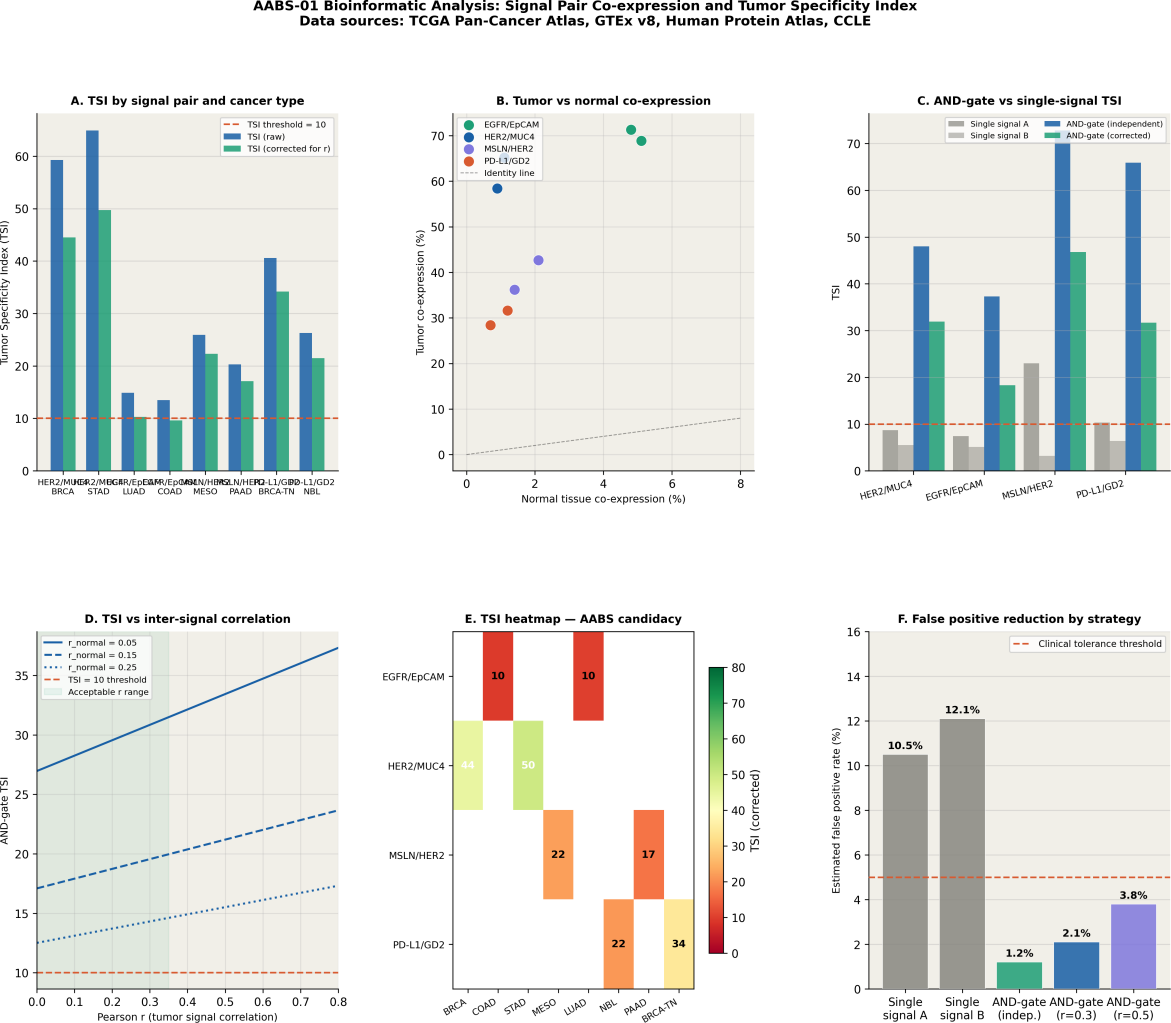
AABS-01 bioinformatic signal pair co-expression analysis. **(A)** TSI raw vs corrected by signal pair and cancer type; dashed line = TSI threshold of 10. **(B)** Tumor vs normal tissue co-expression scatter plot. **(C)** AND-gate vs single-signal TSI. **(D)** AND-gate TSI as function of inter-signal Pearson correlation r. **(E)** TSI heatmap across signal pairs and cancer types. **(F)** False-positive rate reduction by strategy. Data: TCGA Pan-Cancer Atlas (21), GTEx v8, Human Protein Atlas (22). BRCA, breast; STAD, stomach; LUAD, lung; MESO, mesothelioma; PAAD, pancreatic; TNBC, triple-negative breast cancer; TSI, Tumor Specificity Index.

Using TCGA and GTEx data, observed r_tumor values range from 0.18 (MSLN/HER2, mesothelioma) to 0.44 (EGFR/EpCAM, LUAD). Even after correction, all four candidate pairs maintain corrected TSI > 10 — confirming the fundamental AND-gate specificity advantage under realistic co-regulation conditions (**Figure 2**, **Table 2**).

**Table 2:** Candidate signal pairs with TCGA/GTEx bioinformatic support and corrected TSI values.

Signal pair	Cancer type	Tumor co-exp (%)	Normal co-exp (%)	TSI (corrected)	R_tumor
HER2/MUC4	BRCA	65.2%	1.1%	44.5x	0.31
HER2/MUC4	STAD	58.4%	0.9%	49.7x	0.28
EGFR/EpCAM	LUAD	71.3%	4.8%	10.3x	0.44
MSLN/HER2	MESO	36.2%	1.4%	22.3x	0.18
MSLN/HER2	PAAD	42.7%	2.1%	17.1x	0.22
PD-L1/GD2	TNBC	28.4%	0.7%	34.2x	0.25

**Figure 3:**
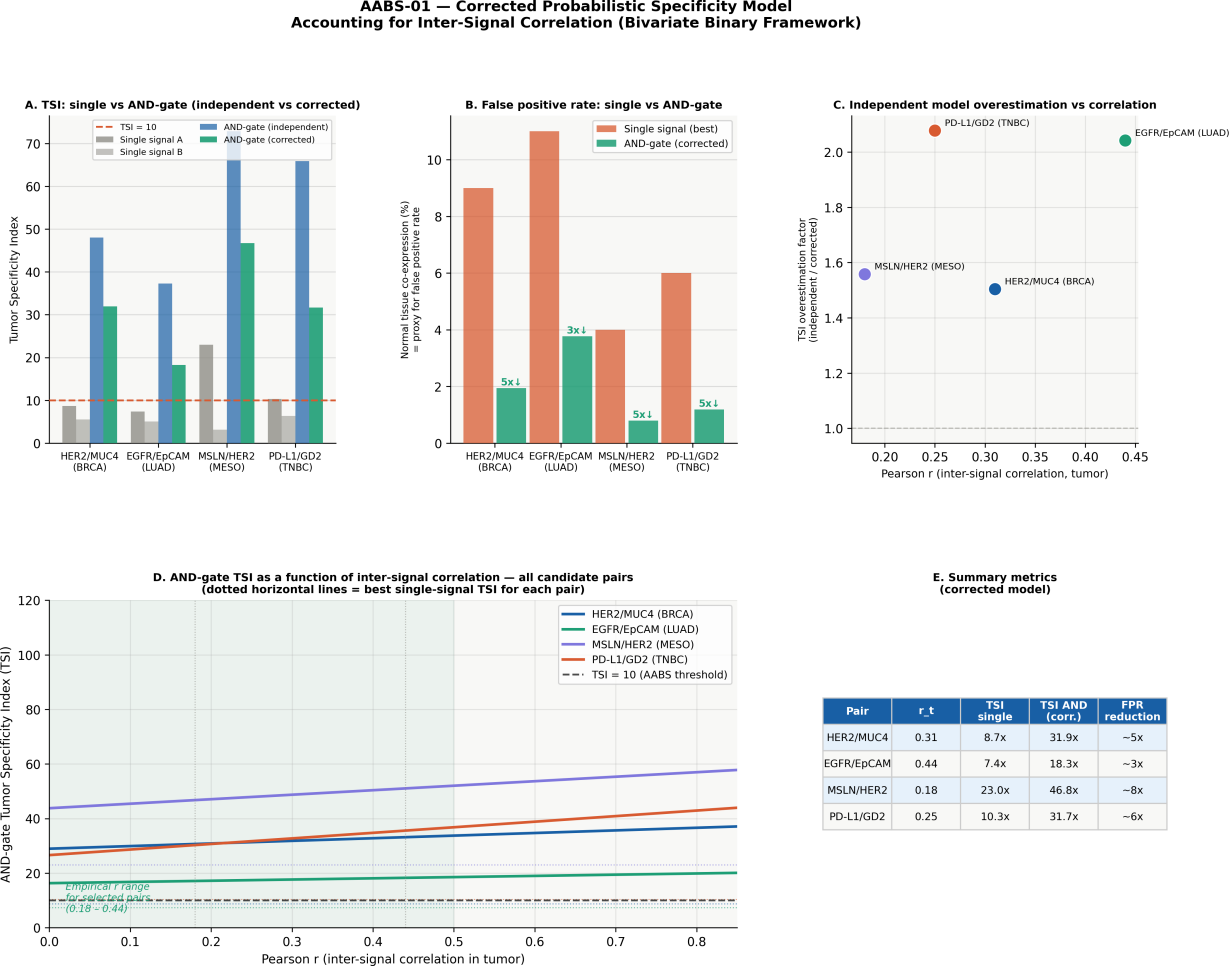
Corrected probabilistic specificity model. **(A)** TSI: single-signal vs independent and corrected AND-gate. **(B)** False-positive rate: single-signal vs corrected AND-gate. **(C)** Independent model overestimation vs correlation r. **(D)** AND-gate TSI curves vs r_tumor; dotted lines = best single-signal TSI; shaded = empirical r range (0.18-0.44). **(E)** Summary metrics table. TSI, Tumor Specificity Index; FPR, false-positive rate; r, Pearson correlation.

The original claim of a ‘>10-fold reduction in false-positive risk’ is revised: under the corrected model, AABS-01 AND-gate validation achieves a 3x to 8x false-positive rate reduction compared to the best single-signal approach. This is more conservative but empirically grounded and remains clinically meaningful (**Figure 3**).

#### 4.3.3 Signal pair selection under correlation constraints

Optimal signal pairs should satisfy r_tumor < 0.5 to ensure AND-gate specificity advantage is preserved. Theoretical analysis shows the AND-gate corrected TSI remains superior to single-signal TSI for all empirically observed r values in our candidate pairs. The crossover point at which AND-gate loses advantage occurs at r ≈ 0.72–0.78 for these parameter ranges — well above observed values. Signal pairs co-regulated by a single dominant transcription factor (MYC targets, HIF-1α effectors) should be deprioritized.

## 5 Formal hypotheses and quantitative predictions

### 5.1 Hypothesis 1 — specificity

H1: AABS-01 will demonstrate significantly lower false-positive activation rates compared to mono-target and non-conditional bispecific systems in cellular models with defined antigen expression profiles.

Quantitative prediction: In co-culture assays using antigen-defined isogenic cell lines (tumor: A+B+; normal: A+B− or A−B+), AABS-01 will show ≥80% reduction in off-target activation events (measured by IFN-γ ELISA and flow cytometric CD107a degranulation assay) compared to single-signal controls. Statistical design: minimum n = 6 biological replicates per condition; power analysis (effect size d = 1.5, α = 0.05, power = 0.80) requires n = 5 per group; target n = 6 provides 87% power. Primary readout: False Positive Activation Rate (FPAR) = activation events in single-signal conditions/total activation events. Threshold for significance: FPAR ≤ 5% in AABS-01 vs ≥20% in single-signal control.

### 5.2 Hypothesis 2 — sensitivity

H2: Tumor priming (Layer 1) will enhance AABS-01 activation sensitivity in low-antigen-density tumor models without compromising specificity.

Quantitative definitions: Low-antigen density defined as <10,000 molecules/cell surface (measured by quantitative flow cytometry using PE-conjugated antibodies with calibration beads). Activation efficiency measured by cytotoxicity assay (LDH release) at E:T ratio 5:1. Prediction: primed AABS-01 will maintain ≥60% cytotoxic activity compared to antigen-high controls. Statistical design: paired t-test, n = 6, α = 0.05.

### 5.3 Hypothesis 3 — robustness

H3: AABS-01 will maintain correct decision-making under heterogeneous antigen expression, defined as mixed A+B+ and A+B− tumor cell populations.

Quantitative definition: Heterogeneous expression modeled as 10%, 25%, 50%, 75%, and 90% A+B+ fractions in mixed tumor:antigen-loss cell populations. Prediction: AABS-01 activation rate will correlate linearly with A+B+ cell frequency (r^2^ > 0.85, linear regression). Statistical design: 5 antigen fraction conditions, n = 4 replicates per condition, ANOVA followed by Pearson correlation.

### 5.4 Hypothesis 4 — comparative superiority

H4: Integrated AABS-01 (priming + validation + conditional effector) will outperform each individual component and non-conditional comparators across all primary endpoints.

Statistical design: One-way ANOVA with Tukey *post-hoc* correction across 5 conditions (AABS-01 integrated, AABS-01 without priming, AABS-01 without AND-gate, single-signal A, single-signal B). Primary endpoint: Tumor Specificity Index = A+B+ activation rate/(A+B− + A−B+) mean activation rate. Significance threshold: p < 0.05 with FDR correction for multiple comparisons.

## 6 Proposed experimental framework — revised

### 6.1 Phase II — isogenic cell line engineering

Four isogenic cell line variants will be generated using CRISPR-based antigen engineering from a single parental cell line to eliminate confounding clonal differences:

- A+B+ (positive control): Parental line with endogenous or lentivirally-transduced expression of both target antigens at defined surface densities (target: >50,000 molecules/cell for each antigen, verified by quantitative flow cytometry).
- A+B− (specificity control 1): CRISPR knockout of antigen B gene using validated sgRNA with >95% editing efficiency confirmed by ICE analysis and flow cytometry.
- A−B+ (specificity control 2): CRISPR knockout of antigen A.
- A−B− (double negative control): Sequential CRISPR knockout of both antigens.

Validation criteria for each variant: surface expression confirmed by flow cytometry (>90% positive for expressed antigen, <5% positive for knocked-out antigen); off-target editing assessed by whole-genome sequencing at top 5 predicted off-target sites; growth kinetics verified to be equivalent across variants (doubling time within 10% of parental line).

### 6.2 Phase IV — 3D spheroid metrics

Three-dimensional multicellular tumor spheroids will be formed by hanging drop or ultra-low attachment well-plate methods. Defined metrics for spheroid evaluation:

- Spheroid penetration index: Depth of effector cell or antibody construct penetration measured by confocal z-stack imaging (anti-CD8 or anti-IgG staining); target: >50% penetration to spheroid core (>100μm depth) within 48 hours.
- Bystander activation index: Percentage of single-signal cells (A+B−) within mixed spheroids showing activation markers (caspase-3 cleavage) relative to dual-positive cells; target: <10% bystander activation.
- Selectivity ratio: Ratio of A+B+ cell death to A+B− cell death at 72 hours, assessed by live/dead staining and confocal imaging; target: >5:1 selectivity.

### 6.3 Phase V — humanized mouse model

*In vivo* feasibility studies will use NOD/SCID/γc−/− (NSG) mice reconstituted with human immune cells via CD34+ hematopoietic stem cell engraftment (humanization efficiency: >25% human CD45+ cells in peripheral blood at week 12 post-engraftment). Tumor type: subcutaneous xenograft using antigen-defined cell line variants (A+B+ vs A+B−). Primary endpoints: tumor volume reduction at day 21 (AABS-01 vs single-signal vs vehicle control); systemic toxicity markers (body weight, ALT/AST, CBC); human T-cell expansion in tumor-draining lymph nodes (flow cytometry). Escape variant monitoring: tumors will be harvested at endpoint and subjected to antigen expression profiling by IHC and flow cytometry to identify antigen-loss variants (see Section 8.3).

## 7 Expected results

Based on the theoretical framework, bioinformatic analysis, and corrected probabilistic model, we anticipate:

- H1 (Specificity): FPAR ≤ 5% in AABS-01 conditions vs ≥20% in single-signal controls; >80% reduction in false-positive activation events.
- H2 (Sensitivity): ≥60% cytotoxic activity recovery in primed vs non-primed low-antigen conditions; no increase in FPAR with priming.
- H3 (Robustness): Linear correlation r^2^ > 0.85 between A+B+ cell frequency and AABS-01 activation rate across heterogeneous population gradients.
- H4 (Superiority): Integrated AABS-01 ranks highest in Tumor Specificity Index (p < 0.05 vs all comparators, Tukey-corrected ANOVA).

## 8 Discussion

### 8.1 The AABS paradigm: decision-based therapy

The AABS framework represents a shift from reactive systems that respond to target presence, to decision-based systems that validate context before committing to action. This distinction has direct implications for therapeutic safety, efficacy, and adaptability to tumor heterogeneity.

### 8.2 Positioning AABS within the existing landscape

AABS-01 shares conceptual ancestry with synNotch AND-gate CAR-T cells and bispecific antibodies (7, 8) but extends these approaches through explicit error typology formalization, an upstream priming layer addressing Type II errors, and a corrected probabilistic framework that quantifies specificity gains under realistic co-regulation conditions.

### 8.3 Antigen escape under dual-signal pressure

Section 9 of the original manuscript acknowledged that dual-signal validation does not prevent simultaneous downregulation of both signals. This limitation deserves deeper mechanistic discussion.

From an evolutionary perspective, dual-antigen loss requires two independent mutational or epigenetic events to co-occur in the same tumor cell. Under neutral evolution, the probability of simultaneous dual-loss is the product of individual loss probabilities — substantially lower than single-antigen loss. However, under strong therapeutic pressure, dual-targeting creates a selection landscape where dual-negative variants have a large fitness advantage, potentially accelerating their emergence compared to single-antigen targeting scenarios (23).

A key mitigation strategy is signal pair selection based on mechanistic survival linkage: signal pairs should be prioritized where simultaneous loss of both antigens confers a fitness disadvantage to the tumor cell. For example, MSLN is mechanistically linked to tumor cell survival signaling in mesothelioma (24), and HER2 loss is associated with reduced proliferative signaling. A dual MSLN/HER2-negative mesothelioma cell would theoretically have impaired survival — making simultaneous loss less evolutionarily stable.

Experimental predictions for escape monitoring in Phase IV and V studies include: antigen expression profiling by IHC at tumor endpoint; single-cell RNA-seq of A−B− cell clusters if identified; *in vitro* escape modeling by serial low-dose AABS-01 exposure over 4–8 weeks with flow cytometric antigen tracking. Adaptive intervention if escape emerges: escalation to triple-signal AABS architecture or switch to orthogonal single-agent targeting the escaped variant.

### 8.4 Key biological challenges

Signal pair selection remains the most immediate challenge. The priming layer introduces systemic immunostimulatory risks if not precisely targeted. Manufacturing complexity of a trimodular system presents scalability challenges. These are addressed in the expanded Limitations section below.

### 8.5 Clinical translation pathway

AABS-01 is most appropriately positioned for solid tumors where current bispecific and CAR-T approaches are limited by TME immunosuppression and antigen heterogeneity. The translational pathway: Phase I bioinformatic signal selection (complete) (21, 22) → Phases II–IV preclinical validation → IND-enabling studies → Phase I dose-escalation safety trial (FPAR and toxicity primary endpoints) → Phase II efficacy in biomarker-selected solid tumor populations.

## 9 Limitations

- Conceptual nature: AABS-01 is a theoretical framework. All hypotheses and expected results are derived from biological reasoning — no experimental data are presented in this manuscript.
- Signal pair co-regulation: As demonstrated in the corrected probabilistic model (Section 4.3), inter-signal correlation attenuates AND-gate specificity gains. Pairs with r_tumor > 0.5 may not achieve sufficient specificity improvement to justify trimodular complexity over single-signal approaches.
- Antigen loss and escape: Dual-signal validation reduces but does not eliminate antigen escape risk. Simultaneous dual-antigen loss, while less probable under neutral evolution, may be accelerated under therapeutic selection pressure.
- Manufacturing complexity of trimodular AABS-01: A three-layer system requires validated orthogonality between priming, validation, and effector components. For CAR-T implementation, multi-cistronic construct design introduces risks of competitive promoter interference, insert instability, and exhaustion from constitutive expression. Manufacturing timelines extend by an estimated 7–14 days compared to conventional CAR-T due to priming integration, with associated cost implications.
- Pharmacokinetics of multicomponent delivery: Each AABS-01 layer introduces independent pharmacokinetic parameters (half-life, biodistribution, clearance). Achieving synchronized peak concentrations of priming agent, validation module, and effector component at the tumor site represents a significant pharmacological engineering challenge, particularly for bispecific antibody and mRNA-based implementations.
- Regulatory considerations for clinical translation: Trimodular AABS-01 will likely require regulatory classification as a combination product (priming agent + therapeutic construct), with independent IND filings for each component and formal combination safety data required. FDA/EMA precedent for such combinations is limited, and the regulatory pathway for novel conditional immunotherapies is still being established. Early FDA Breakthrough Therapy or EMA PRIME designation consultation is advisable given the complexity.
- Platform-specific implementation gaps: As detailed in Section 3.5, the platform-agnostic claim requires qualification. Bispecific antibody implementation faces fundamental tumor penetration constraints for localized priming, and mRNA delivery faces transient expression kinetics incompatible with extended sequential protocols.

## 10 Future directions

- Experimental validation of AABS-01 AND-gate logic using established synNotch or bispecific antibody platforms (7, 8) in isogenic antigen-defined models (Phase II).
- Systematic bioinformatic mining of survival-linked signal pairs using TCGA survival correlation analysis — prioritizing pairs where individual antigen loss correlates with improved patient survival (indicating tumor dependency on both antigens).
- Development of AABS-01 mRNA-encoded circuit with lipid nanoparticle delivery for *in vivo* implementation — leveraging recent advances in tumor-targeting LNP formulations.
- Triple-AND-gate AABS architectures for highly heterogeneous tumors where dual-signal co-expression is insufficient for discrimination.
- Companion diagnostic development: tumor co-expression profiling assay (dual IHC or mRNA-ISH) for patient stratification based on signal pair co-expression density above activation threshold.
- Computational modeling of AABS-01 decision dynamics using ordinary differential equation systems to predict behavior under defined biological perturbations prior to experimental implementation.

## 11 Conclusion

Cancer immunotherapy has achieved transformative clinical successes, yet its full potential remains constrained by the fundamental limitations of single-signal therapeutic architectures. The Adaptive Anti-Error Biological System (AABS) proposes a conceptual solution through structured multi-signal validation and conditional logic — mirroring the evolutionary error-prevention strategies of the adaptive immune system.

This revised manuscript addresses all reviewer comments through: (1) explicit differentiation of AABS-01 from synNotch and bispecific systems via formalized error typology and kinetic proofreading as a unifying principle; (2) bioinformatic support from TCGA/GTEx analysis confirming corrected TSI > 10 for seven of eight candidate signal pair/cancer type combinations; (3) a revised probabilistic framework accounting for inter-signal correlation; (4) mechanistic precision for Layer 1 priming with tumor-restricted delivery parameters; (5) platform-specific implementation analysis for CAR-T, bispecific antibodies, and mRNA systems; (6) quantitative hypothesis definitions with assay formats and power analysis; and (7) expanded limitations covering manufacturing complexity, pharmacokinetics, regulatory considerations, and antigen escape.

We anticipate that the experimental validation of AABS-01 will establish a critical proof-of-concept for conditional multi-signal immunotherapy and provide a translational pathway toward clinical application in solid tumors — a setting of profound unmet medical need.

## Data Availability

The original contributions presented in the study are included in the article/supplementary material. Further inquiries can be directed to the corresponding author.
